# Engineering inter-kingdom adrenergic signaling in commensals couples host stress hormone sensing to programmable biological outputs

**DOI:** 10.1371/journal.pbio.3003926

**Published:** 2026-08-25

**Authors:** Santosh Kumar Srivastava, Guo Wei Foo, Haosheng Shen, Yuanzhi He, Kwok Soon Wun, In Young Hwang, Michael S. Goodson, Nikhil Aggarwal, Matthew Wook Chang

**Affiliations:** 1 NUS Synthetic Biology for Clinical and Technological Innovation (SynCTI), National University of Singapore, Singapore, Singapore; 2 Synthetic Biology Translational Research Programme, Yong Loo Lin School of Medicine, National University of Singapore, Singapore, Singapore; 3 National Centre for Engineering Biology (NCEB), Singapore, Singapore; 4 School of Healthcare Science and Engineering (SHINE), Vellore Institute of Technology, Vellore, India; 5 Department of Biochemistry, Yong Loo Lin School of Medicine, National University of Singapore, Singapore, Singapore; 6 United States Air Force Research Laboratory, Wright Patterson Air Force Base, Dayton, Ohio, United States of America; Zhejiang University, CHINA

## Abstract

Host stress is associated with elevated catecholamine neurohormones that influence gut physiology and host–microbe interactions, yet how bacterial systems detect and interpret these signals remains incompletely understood. Enteric pathogens exploit inter-kingdom adrenergic signaling to sense host-derived norepinephrine and epinephrine, but whether such pathways can be rationally rewired to produce predictable, programmable outputs has not been systematically explored. Here, we reconstitute adrenergic signaling in *Escherichia coli* Nissle 1917 by repurposing the enterohemorrhagic *E. coli* QseBC two-component system. Transcriptomic profiling revealed robust catecholamine-dependent activation of QseBC-regulated pathways in the engineered strain. Guided by these data, we redesigned a QseBC-responsive promoter through rational truncation, sigma-factor replacement, and optimization of QseBC expression, generating a synthetic promoter with enhanced sensitivity and dose-dependent responsiveness to stress hormones. Structure-guided mutagenesis of the QseC sensor kinase identified key residues required for catecholamine recognition, providing mechanistic insight into adrenergic hormone sensing. To demonstrate functional signal transduction beyond transcriptional reporting, we coupled the sensing module to a secretion cassette encoding a corticotropin-releasing factor (CRF) receptor antagonist as a model bioactive output and validated bioactivity in vitro. Together, this work elucidates principles governing bacterial stress hormone sensing and demonstrates how inter-kingdom signaling pathways can be engineered to yield programmable biological outputs.

## Abstract 2

Engineering microbial systems capable of sensing host-derived stress hormones has proved technically challenging. This study reconstitutes QseBC-mediated adrenergic sensing in Escherichia coli to create a catecholamine-responsive engineered probiotic strain capable of sensing host stress and coupling this detection to the secretion of bioactive therapeutic molecules.

## Introduction

Chronic psychological stress has emerged as a significant global health challenge, profoundly impacting both mental and physical well-being [1–3]. A key outcome of prolonged stress is the disruption of the gut–brain axis, a bidirectional communication system linking the central nervous system with the gastrointestinal tract [4]. Stress activates the hypothalamic–pituitary–adrenal (HPA) axis and the sympathetic nervous system, leading to elevated levels of catecholamine neurohormones, particularly norepinephrine (NE) and epinephrine (Epi) [2,5]. These neurohormones function as reliable biomarkers of stress and play critical roles in modulating host–microbe interactions within the gut [6,7]. In parallel, chronic stress induces sustained production of corticotropin-releasing factor (CRF) and its related neuropeptides (Ucn1, Ucn2, and Ucn3) in both the central nervous system and intestinal epithelial cells [8–10]. These stress-induced perturbations of gut–brain communication can profoundly affect gut function, contributing to the onset or exacerbation of gut disorders [9,10].

Despite growing recognition of neuroendocrine regulation in the gut, engineering microbial systems capable of sensing host-derived stress hormones and converting these signals into interpretable transcriptional outputs remains a challenge. While microbial-based “sense-and-respond” approaches, in which engineered microbes convert host signals into regulated biological responses, offer a potential route to interrogate and modulate host–microbe communication in situ, construction of microbial biosensors for human hormones is complicated by ligand instability, interference from endogenous bacterial regulatory networks, and the need for robust signal transduction under gut-like conditions. Addressing these challenges requires both suitable hormone-sensing modules and rationally designed regulatory architectures that yield predictable outputs.

Notably, several enteric pathogens have evolved mechanisms to sense host catecholamines as environmental cues. Enterohemorrhagic *Escherichia coli* O157:H7 (EHEC) exploits inter-kingdom adrenergic signaling to detect NE and Epi and regulate motility, biofilm formation, and virulence gene expression [11–13]. Central to this response is the QseBC two-component system, in which the sensor kinase QseC detects catecholamines and activates the response regulator QseB to coordinate downstream transcriptional programs [11,14]. This natural signaling pathway illustrates a broader biological principle: bacteria can interpret host neurohormones and translate them into coordinated gene expression responses [5,13].

Here, we sought to repurpose this inter-kingdom adrenergic signaling mechanism as an engineering framework for constructing hormone-responsive microbial systems. We selected *Escherichia coli* Nissle 1917 (EcN), a clinically validated probiotic strain, as the chassis organism. Although EcN harbors homologs of *qseBC*, naturally occurring mutations render its endogenous system incapable of functional adrenergic sensing. To reconstitute catecholamine responsiveness, we expressed the EHEC *qseBC* operon in EcN and performed transcriptomic profiling following NE exposure to assess system functionality and identify QseBC-responsive regulatory pathways [15–19].

Among the pathways activated by adrenergic signaling, the flagellar biosynthesis master regulator operon *flhDC* plays a central role in integrating environmental signals to coordinate motility and stress-adaptive responses in EHEC through hierarchical regulation of downstream flagellar genes [20]. In heterologous contexts, however, *flhDC* regulation is subjected to interference from global transcriptional regulators and sigma-factor–dependent control mechanisms. To address these constraints, we redesigned the *flhDC* promoter architecture through truncation, sigma-factor replacement, and tuning of QseBC expression to generate a robust adrenergic-responsive transcriptional output.

To probe the molecular basis of catecholamine sensing, we combined structural modeling of the QseC periplasmic domain with site-directed mutagenesis to examine residues involved in ligand recognition, extending prior observations that QseC-mediated signaling underlies NE-dependent transcriptional regulation in EHEC. This approach enabled mechanistic interrogation of adrenergic hormone detection within the engineered system.

Finally, to demonstrate functional signal transduction beyond transcriptional reporting, we coupled the optimized sensing module to a secretion cassette encoding the CRF receptor antagonist peptide rCRF(9–41), used here as a model bioactive output. Functional validation was performed using in vitro macrophage and intestinal epithelial cell models. Together, these experiments establish an engineering-driven framework for rewiring inter-kingdom adrenergic signaling and converting host stress hormone detection into programmable biological outputs [21].

## Results

### Reconstitution of adrenergic signaling restores catecholamine-responsive transcription in *E. coli* Nissle 1917

**Fig 1:**
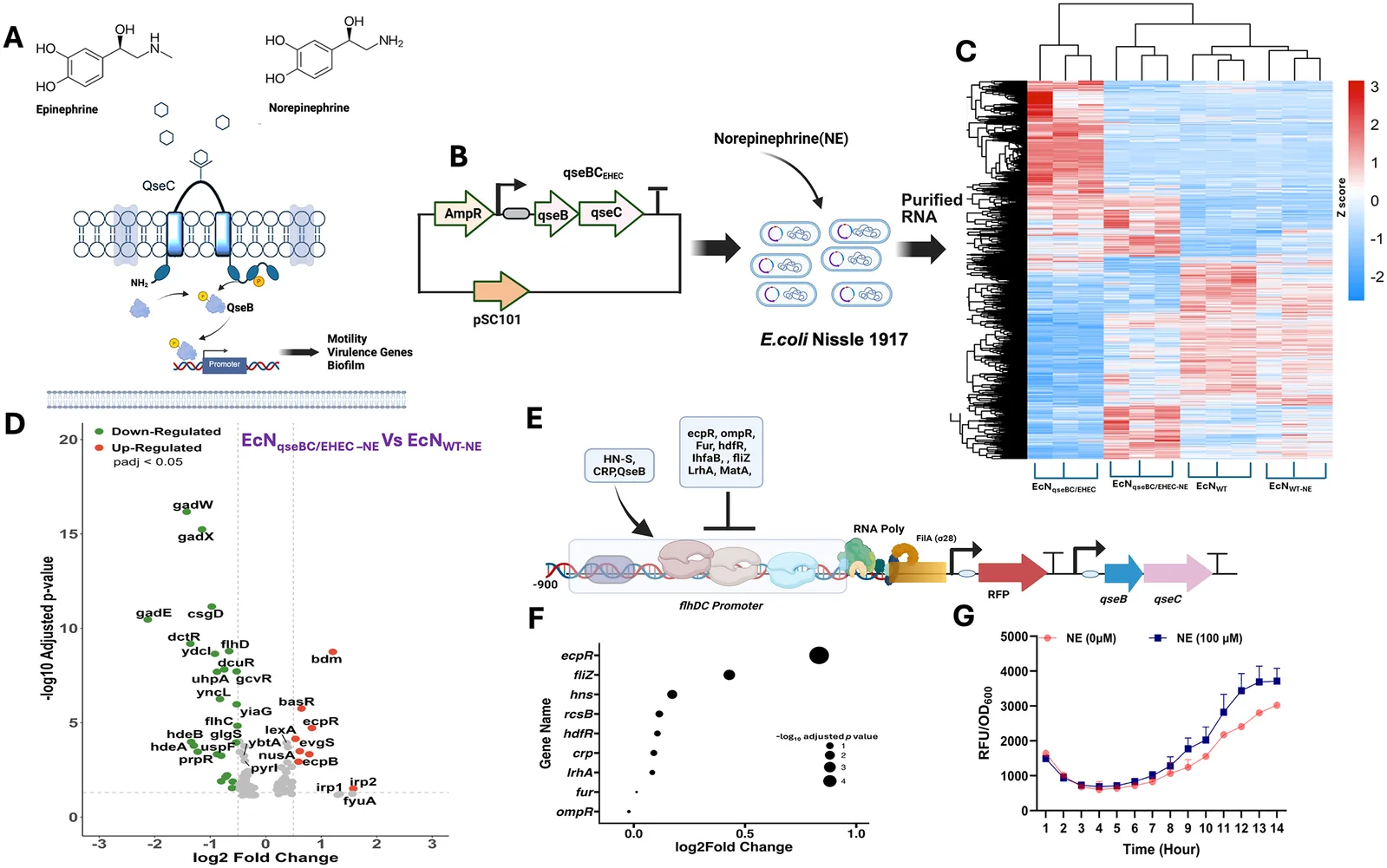
Transcriptomic profiling and characterization of adrenergic signaling in *EcN.* **(A)** Schematic overview of the QseBC-mediated adrenergic signaling pathway in EHEC. QseC detects NE and Epi and activates the response regulator QseB to drive transcriptional responses. **(B)** Genetic strategy for heterologous expression of the EHEC *qseBC* operon in EcN using a low copy pSC101 plasmid under a constitutive promoter (J23111), followed by NE exposure. RNA was isolated from untreated EcN_WT_, NE-treated EcN_WT_, and NE-treated EcN expressing *qseBC*_EHEC_. **(C**) Hierarchical clustering heatmap of gene expression profiles highlighting NE-dependent transcriptional changes across the indicated treatment groups. **(D)** Volcano plot highlighting the selected upregulated and downregulated genes in EcN_*qseBC*-EHEC-NE_ vs EcN_WT-NE_, emphasizing the specific genes regulated by the QseB in response to NE exposure**.** RNA-seq–derived differentially expressed genes (DEGs), selected using thresholds of padj < 0.05 and |log_2_(fold change) > 0.5. Differential expression analysis was performed in R using the DESeq2 package. **(E)** Schematic representation of potential regulators interacting with the *flhDC*_*EHEC*_ operator that may either constrain or facilitate activation of the *flhDC*_*EHEC*_ operon **(F)** Bubble plot summarizing transcriptional changes of regulatory factors associated with the *flhDC*_*EHEC*_ regulon following NE induction in the engineered EcN_*qseBC*-EHEC_ strains compared to EcN_WT-NE_. **(G)** Evaluation of NE sensitivity using the *flhDC*_EHEC_ promoter fused to RFP in EcN, with fluorescence intensity normalized to OD₆₀₀ after exposure to 100 µM NE. Data are presented as mean ± SEM (*n* = 3). The data underlying this Figure can be found in S1 Data. Raw transcriptomic data can be found under BioProject accession number PRJNA1467173. Created in BioRender. Aggarwal, N. (2026) https://BioRender.com/z371w74.

To determine whether adrenergic signaling could be reconstituted in *E. coli* Nissle 1917 (EcN), we heterologously expressed the enterohemorrhagic *E. coli* (EHEC) *qseBC* operon, which encodes the catecholamine-responsive sensor kinase QseC and its cognate response regulator QseB (Fig 1A). Although EcN harbors homologs of qseBC with high sequence similarity to those of EHEC (~97% identity), bioinformatic analysis revealed naturally occurring mutations in both qseC and qseB that are predicted to impair adrenergic sensing (S1 and S2 Figs). To restore catecholamine responsiveness, we expressed the EHEC qseBC cassette under a synthetic constitutive promoter (J23111) on a low-copy-number plasmid (pSC101) (Fig 1B).

To assess transcriptional responses to norepinephrine (NE), we performed RNA sequencing on four conditions: untreated wild-type EcN (EcN_WT_), NE-treated EcN_WT_, EcN expressing the *qseBC* operon (EcN_*qseBC*–EHEC_) and NE-treated EcN_*qseBC*–EHEC_. NE exposure elicited minimal transcriptional changes in wild-type EcN, indicating weak endogenous adrenergic responsiveness (S3A Fig). In contrast, EcNqseBC–EHEC exhibited robust NE-dependent transcriptional reprogramming, with distinct expression profiles evident in both heatmap and volcano plot analyses, when compared to untreated EcN expressing the qseBC operon (Figs 1C and S3A).

To distinguish NE-dependent effects from basal qseBC expression, we additionally compared uninduced EcNqseBC–EHEC with the corresponding NE-induced condition, which revealed widespread transcriptional reprogramming and substantial changes in gene expression (S4 Fig). Overall, differential expression analysis showed that NE-induced QseBC signaling preferentially regulated genes associated with flagellar biogenesis, quorum sensing, stress adaptation, and biofilm formation [13,22] (Figs 1D and S3B). These pathways mirror transcriptional programs previously reported for adrenergic signaling in EHEC, confirming that the heterologously expressed *qseBC* operon is functional in the EcN background and capable of transmitting catecholamine-dependent signals.

Among the NE-responsive pathways, genes within the flagellar biosynthesis regulon were prominently represented. This regulon is hierarchically controlled by the master regulator operon *flhDC*, which integrates environmental inputs to coordinate motility and stress-adaptive responses in EHEC (S3C Fig). However, despite broad activation of downstream flagellar genes, transcription of the *flhDC* operon itself was only weakly induced by NE in EcN_*qseBC*–EHEC_ (S3B Fig).

The limited responsiveness of *flhDC* in this heterologous context is consistent with interference from global transcriptional regulators and sigma-factor–dependent control mechanisms known to act on the *flhDC* promoter, including H-NS, RcsB, EcpR, HdfR, and FliZ [22] (Fig 1E and 1F). In addition, sequence divergence in the endogenous EcN flagellar promoters may further reduce QseB binding affinity relative to their EHEC counterparts (S3D Fig).

To identify adrenergic-responsive promoter elements suitable for biosensor construction, we therefore evaluated flagellar gene promoters derived from EHEC rather than EcN. The *flhDC*, *flgBCDEF*, and *flgGHIJ* promoters from EHEC were fused to a red fluorescent protein (RFP) reporter and co-expressed with *qseBC*_EHEC_ in EcN. Promoter activity was assessed under microaerobic conditions sealed tube or sealed plate in Dulbecco’s Modified Eagle Medium (DMEM), which enhances QseBC-dependent signaling in EHEC [23]. Among the promoters tested, only the *flhDC*_EHEC_ promoter exhibited measurable NE-dependent induction (Fig 1G), whereas the *flgBCDEF*_EHEC_ and *flgGHIJ*_EHEC_ promoters showed no detectable responsiveness (S3E and S3F Fig).

Together, these results establish that heterologous expression of the EHEC *qseBC* operon restores catecholamine-responsive transcription in EcN and identify *flhDC*_EHEC_ as a QseBC-regulated promoter with intrinsic but constrained NE responsiveness. This finding provided the basis for subsequent promoter engineering to enhance adrenergic signal transduction.

### Promoter truncation reduces regulatory interference and enhances adrenergic responsiveness of *flhDC*

**Fig 2:**
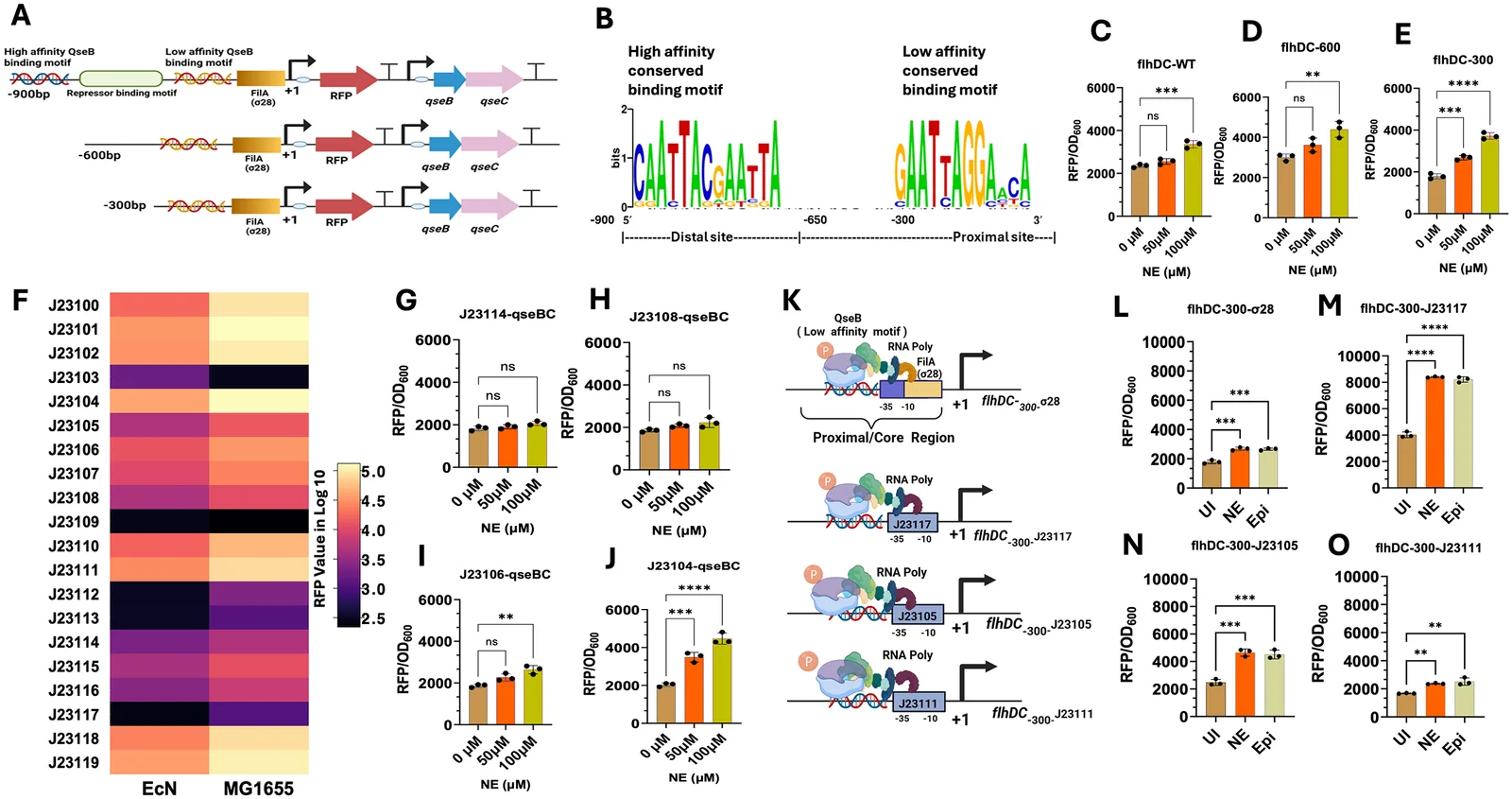
Truncation of *flhDC*_EHEC_ promoter and synthetic rewiring enhances QseBC-mediated adrenergic sensing. **(A)** Schematic showing the truncation of the wild-type *flhDC*_*EHEC*_ promoter to generate *flhDC*_*EHEC*-300_ and *flhDC*_*EHEC-*600_ variants enabling reduced regulatory complexity and increased sensitivity to adrenergic signaling. (**B)** WebLogo representation of conserved high- and low-affinity QseB-binding motifs together with the genomic organization of proximal and distal QseB-binding regions within the native *flhDC*_*EHEC*_ promoter. **(C-E)** Quantification of NE-responsive transcriptional activity of full-length and truncated *flhDC*_*EHEC*_ variants fused to RFP following exposure to 50 μM and 100 μM NE **(F)** Heatmap comparing relative activities of Anderson synthetic promoters in EcN and *E. coli* MG1655, highlighting strain-dependent differences in promoter strength. (**G-J)** Effect of *qseBC*_EHEC_ expression stoichiometry on NE signal transduction. Expression of the *qseBC*_EHEC_ operon was tuned using synthetic promoters of defined strengths, and NE dependent activation of the *flhDC*_EHEC-300_ promoter was quantified by normalized RFP fluorescence following exposure to 50 μM and 100 μM NE. **(K)** Schematic representation of *flhDC_−300_* promoter variants in which the native σ^28^-dependent region of *flhDC_−300_* was replaced with synthetic σ70 promoters of defined strengths (J23117, J23105, J23111) to overcome environmental regulation and enhance transcriptional stability. **(L–O)** Fluorescence-based reporter assay showing the activity of engineered *flhDC*-_EHEC_ promoter variants in response to 50 μM NE or Epi. The J23117 variant showed the highest induction and lowest variability. (All Data are presented as mean ± SEM (*n* = 3). One-way ANOVA followed by Tukey’s multiple comparisons test was used for the statistical analysis; *p < 0.05, **p < 0.01, ***p < 0.001, ****p < 0.0001 vs. uninduced (0µM NE). The data underlying this Figure can be found in S1 Data. Created in BioRender. Aggarwal, **N.** (2026) https://BioRender.com/z371w74.

To determine whether regulatory complexity limits adrenergic responsiveness of the *flhDC*_EHEC_ promoter, we examined its native architecture in light of prior studies [14,20,22]. The *flhDC*_EHEC_ promoter contains a σ^28^-binding site in its proximal region, while the central and distal regions harbor multiple binding sites for global transcriptional regulators as well as phosphorylated QseB, including both high- and low-affinity motifs [20,22,24] (Fig 2A and 2B).

To reduce potential interference from competing regulatory inputs while preserving QseB responsiveness, we generated truncated *flhDC*_EHEC_ promoter variants. The *flhDC*_EHEC-600_ variant retains several regulatory binding motifs in addition to the low-affinity QseB-binding site, whereas the *flhDC*_EHEC-300_ variant preserves only the low-affinity QseB-binding site together with the core σ^28^-dependent regulatory region (Fig 2A). Each promoter variant, along with the full-length *flhDC*_EHEC_ promoter, was fused to a RFP reporter on a low-copy-number plasmid (pSC101).

Promoter activity was assessed under microaerobic conditions following exposure to NE. Compared to the full-length and *flhDC*_EHEC-600_ promoters, the *flhDC*_EHEC-300_ variant exhibited significantly enhanced NE responsiveness at both 50 and 100 μM NE (Fig 2C–2E). These results indicate that removal of distal regulatory elements increases adrenergic sensitivity, consistent with the presence of competing regulators that constrain QseB-mediated transcriptional activation in the native promoter context.

### QseBC expression level and translational coupling govern catecholamine sensing

To determine how QseBC abundance influences catecholamine sensing, we examined the effect of *qseBC* expression level on NE-dependent transcriptional output. Because promoter activity can vary substantially between *E. coli* strains, we first assessed a panel of Anderson synthetic promoters in both EcN and the laboratory strain MG1655 (Figs 2F and S5). Despite high genomic similarity, promoter strengths differed markedly between strains, underscoring the strain-specific nature of transcriptional control.

Based on promoter activity in EcN, we selected synthetic promoters spanning a range of strengths to drive the expression of EHEC *qseBC* operon, together with an engineered RBS containing a canonical AGGAG Shine–Dalgarno motif while preserving native operon architecture. The engineered RBS was designed using the Salis Lab RBS Calculator and yielded a predicted translational initiation rate of ~10,000, nearly 10-fold higher than the native *qseBC* RBS. These constructs were combined with the *flhDC*_EHEC-300_-RFP reporter. Increasing *qseBC* expression enhanced NE responsiveness, with J23104 producing the highest reporter induction among the promoter configurations tested, whereas weaker promoters yielded diminished responses (Fig 2G–2J). These results indicate that QseBC expression level is a key determinant of sensor sensitivity.

In native EHEC, *qseB* and *qseC* are encoded within a single operon and are translationally coupled via overlapping stop and start codons (S6A Fig). To assess whether this organization influences sensing performance, we decoupled *qseB* and *qseC* expression by placing each gene under independent promoters (S6B Fig). While QseB and QseC protein expression was detectable by SDS–PAGE (S6C Fig), decoupling their expression resulted in a marked reduction in NE responsiveness compared to the operon-encoded configuration (S6D and S6E Fig).

The reduced responsiveness observed upon decoupling is consistent with prior reports that translational coupling ensures coordinated expression and functional stoichiometry of two-component system components. Together, these results demonstrate that both QseBC expression level and translational coupling are critical for efficient catecholamine sensing and identify the *flhDC*_EHEC-300_ promoter combined with operon-encoded *qseBC* driven by J23104 as the optimal configuration for subsequent analyses.

### Sigma-factor replacement and QseB motif engineering yield a synthetic catecholamine-responsive promoter

To reduce context-dependent variability in adrenergic signaling, we next examined the role of sigma-factor dependence in regulating *flhDC* transcription. In EHEC, transcriptional initiation at the *flhDC*_EHEC_ promoter is mediated in part by σ^28^, whose activity is modulated by the anti-sigma factor FlgM under non-permissive conditions [25]. The truncated *flhDC*_EHEC-300_ promoter retains this native σ^28^-binding region which may constrain promoter activity under conditions where σ^28^ availability is limited.

We therefore decoupled adrenergic responsiveness from σ^28^-dependent regulation by replacing the native σ^28^-binding site in the *flhDC*_EHEC-300_ promoter with synthetic σ^70^-dependent promoters of defined strengths (Fig 2K). Among the variants tested, substitution with the weakest σ^70^ promoter (J23117) produced the most robust and reproducible induction in response to NE and epinephrine (Epi), whereas stronger σ^70^ promoters exhibited reduced dynamic range and increased variability (Fig 2L–2O). Synthetic promoter activity is highly context dependent, with local sequence architecture and spacing constraints substantially influencing signaling output [26]. Consistent with this structural sensitivity, replacing the native σ^28^ region within the truncated *flhDC*_-300_ promoter altered promoter behavior and deviated from canonical Anderson promoter rankings.

**Fig 3:**
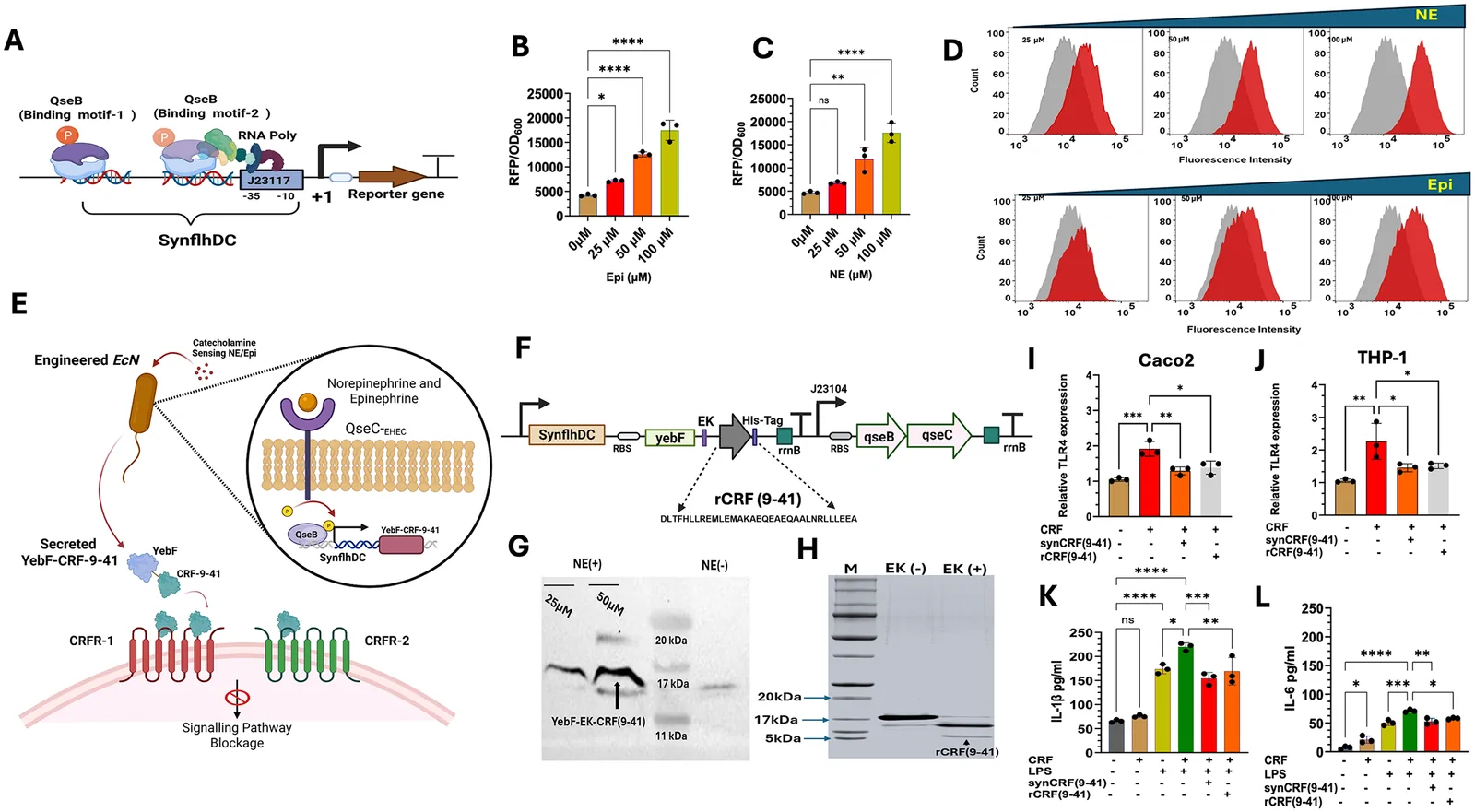
A synthetic QseB-responsive promoter enables dose-dependent catecholamine sensing and controlled secretion of a CRF antagonist. **(A)** Design of a synthetic promoter (SynflhDC) incorporating both high and low-affinity QseB binding sites to enhance adrenergic responsiveness. **(B, C)** Quantification of normalized RFP fluorescence demonstrated a clear dose-dependent activation pattern with NE and Epi (25, 50, and 100 μM). **(D)** Flow cytometry analysis showing population-level fluorescence shifts corresponding to graded NE/Epi responsive promoter activation. **(E)** Illustration depicting engineered EcN detecting NE and Epi via the QseBC two-component system, resulting in conditional expression and secretion of the defined biological output rCRF(9–41) **(F)** Schematic of an NE/Epi-responsive genetic circuit coupled to a YebF–EK–rCRF (9–41) gene cassette. **(G**) Western blot analysis of extracellular fractions showing NE dependent secretion of the YebF–EK–CRF(9–41) fusion protein, detected using an anti-His HRP–conjugated antibody. SDS–PAGE analysis showing EK cleavage of the purified YebF–EK–CRF(9–41) fusion protein to release the rCRF(9–41) peptide. **(I, J)** qRT-PCR analysis showing modulation of CRF (100 nM)–mediated TLR4 expression in THP-1 macrophages and Caco-2 cells following pretreatment with either recombinant [rCRF(9–41)] or synthetic [synCRF(9-41)] antagonist (500 nM). **(K, L)** Quantification of IL-6 and IL-1β in differentiated THP-1 macrophages treated with CRF (100 nM) and LPS (1 µg/mL), alone or in combination, following pretreatment with either rCRF(9-41) or synCRF(9-41) antagonist (500nM). Data are presented as mean ± SEM (*n* = 3) and were analyzed by one-way ANOVA with Tukey’s multiple-comparison test (*p < 0.05, **p < 0.01, ***p < 0.001, ****p < 0.0001). The data underlying this Figure can be found in S1 Data and S1 Raw Images. Created in BioRender. Aggarwal, N. (2026) https://BioRender.com/z371w74.

Catecholamine responsiveness was further enhanced by introducing both high- and low-affinity phosphorylated QseB-binding sites at defined positions upstream of the core promoter (Fig 3A). This design was intended to promote cooperative QseB binding and enable graded transcriptional activation. To assess the contribution of motif architecture, we additionally generated a construct containing only the low-affinity QseB-binding site (S7A Fig). Compared with the dual-motif design, the low-affinity-only construct exhibited limited responsiveness across 25–100 μM NE and Epi concentrations (S7B and S7C Fig), supporting a role for combined high- and low-affinity motifs in enhancing catecholamine-responsive transcriptional activation.

The dual-motif engineered promoter, designated SynflhDC, exhibited detectable activation at 25 μM NE and showed progressively enhanced activity at higher concentrations of NE and Epi (Fig 3B and 3C). Additionally, the engineered strain exhibited a similar growth profile to the wild-type EcN strain (S8A Fig). Flow cytometry analysis confirmed population-wide fluorescence shifts consistent with enhanced transcriptional output (Fig 3D). Dose–response analysis further revealed concentration-dependent activation of the engineered sensor, with measurable induction observed from approximately 25 μM, followed by progressive signal enhancement and saturation at higher ligand concentrations (S8B and S8C Fig). This response range is consistent with previously reported QseBC-based catecholamine sensing systems, which typically operate in the low-to-mid micromolar range under in vitro conditions [14,27]. We also evaluated SynflhDC responsiveness to the quorum-sensing signal AI-3 and compared its activation with NE across a 25–100 μM concentration range. Although the engineered system responded to both ligands, NE consistently elicited stronger activation than AI-3, indicating preferential responsiveness to catecholamine signaling under the tested conditions (S8D Fig).

To investigate the molecular basis of catecholamine-responsive signaling by the QseC sensor kinase, we performed structure-guided mutagenesis focused on the predicted periplasmic sensing domain. As no experimentally resolved structure of QseC is currently available, ligand docking analyses were performed using AlphaFold-predicted structural models restricted to the putative sensor domain (residues 34–156). Docking of norepinephrine (NE) and epinephrine (Epi) identified a shared set of candidate interaction residues, including Thr72, Asn74, Asp83, and Asp101, predicted to contribute to ligand-responsive signaling (S9A and S9B Fig).

To evaluate their functional relevance, candidate residues were subjected to site-directed mutagenesis followed by reporter-based analysis of catecholamine responsiveness. Among the variants tested, substitution of Asn74 with alanine (N74A) markedly reduced catecholamine-responsive signaling, whereas D83A and D101A produced partial reductions in activity and T72A showed no detectable effect (S9C–S9G Fig). Given that QseBC signaling can also be activated by the quorum-sensing autoinducer AI-3, we additionally assessed AI-3–responsive signaling in wild-type QseC and the N74A mutant. Reduced activation of the N74A variant following AI-3 stimulation suggests that Asn74 contributes to QseC-mediated signal transduction rather than acting as a selective catecholamine-sensing determinant (S9D Fig).

To further exclude the possibility that the observed signaling defects resulted from altered protein stability or defective membrane localization, we performed preliminary biochemical analysis of membrane-associated QseC variants. Comparable membrane-associated expression of QseC WT and mutant proteins suggested that the reduced signaling phenotypes are unlikely to arise from gross protein misfolding or impaired membrane localization (S10 Fig). However, definitive elucidation of the molecular mechanism underlying QseC-mediated catecholamine sensing will require future structural and direct ligand-binding studies, which are beyond the scope of the present study.

### Catecholamine sensing can be coupled to regulated secretion of a bioactive peptide

To test whether adrenergic sensing could be coupled to a defined biological output, we linked the optimized catecholamine-responsive promoter to a secretion cassette encoding the CRF receptor antagonist peptide rCRF(9–41), used here as a model bioactive output. CRF(9–41) is a well-characterized competitive antagonist of CRF1 receptors and is widely used to probe CRF-mediated signaling pathways [18,28].

To implement a sense-and-respond circuit, we engineered EcN to detect NE/Epi and conditionally secrete the CRFR1 antagonist rCRF(9–41) (Fig 3E). To enable extracellular peptide release, rCRF(9–41) was fused to the *E. coli* secretion carrier YebF via an enterokinase cleavage site (Fig 3F). Engineered EcN strains harboring the optimized sensing module and the YebF–rCRF(9–41) cassette were exposed to NE under microaerobic conditions at concentrations of 25 μM and 50 μM, which fall within the upper physiological range reported for the gut during stress and enteric infection [11,29]. Western blot analysis of concentrated culture supernatants confirmed NE-dependent secretion of the fusion protein following induction with 25 μM and 50 μM NE (Fig 3G). To further quantify peptide production under non-concentrated conditions, secreted rCRF(9–41)-HisTag levels were measured directly from culture supernatants using a HisTag ELISA assay, revealing secretion levels of approximately 65–84 nM under the tested conditions (S8E Fig). Following purification and enterokinase treatment, the expected rCRF(9–41) fragment was released (Fig 3H), confirming correct processing of the secreted product.

NE concentrations in the gut microenvironment are difficult to quantify and may vary substantially under conditions of stress and inflammation [5,12,30,31]. Under these conditions, rCRF (9–41) secretion was reproducibly induced by NE exposure, demonstrating that the engineered genetic circuit converts catecholamine detection into regulated peptide secretion.

### Secreted rCRF(9–41) modulates CRF-dependent inflammatory and barrier responses in vitro

To assess whether the secreted bioactive peptide produced by the engineered sensing module retained functional activity, we performed in vitro analyses using purified recombinant rCRF(9–41) derived from engineered EcN. Direct co-culture of engineered bacteria with mammalian cells was not pursued due to instability of catecholamines under standard cell culture conditions and their rapid degradation by catechol-O-methyltransferase and monoamine oxidases expressed by mammalian cells [32]. Use of purified peptide enabled controlled evaluation of bioactivity independent of these constraints.

Purified YebF–rCRF(9–41) fusion protein was processed by enterokinase to release the active peptide and applied to human THP-1 macrophages and Caco-2 intestinal epithelial cells. Because CRF has been shown to prime innate immune responses through modulation of Toll-like receptor 4 (TLR4) signaling [33], we first examined the effect of rCRF(9–41) on CRF-induced TLR4 expression. CRF treatment increased TLR4 mRNA levels in both cell types, whereas pretreatment with rCRF(9–41) or a synthetic CRF antagonist [synCRF(9-41)] significantly attenuated this response (Fig 3I and 3J). To exclude the possibility that the observed effects resulted from YebF itself or purification-associated contaminants, purified YebF lacking the rCRF(9–41) cargo was processed under identical secretion, purification, and enterokinase-treatment conditions. Treatment with purified YebF alone did not suppress CRF-induced TLR4 expression in either THP-1-derived macrophages or Caco-2 cells (S11A and S11B Fig), supporting that the observed effects are specifically attributable to rCRF(9–41).

We next examined whether rCRF(9–41) could modulate inflammatory responses under conditions of combined neuroendocrine and microbial stimulation. In differentiated THP-1 macrophages, CRF alone had minimal effect on IL-6 and IL-1β expression, whereas co-stimulation with CRF and lipopolysaccharide (LPS) increased pro-inflammatory cytokine expression (Figs 3K, 3L, and S12). Pretreatment with rCRF(9–41) reduced cytokine induction to levels comparable to those observed with a synthetic CRF antagonist, indicating effective antagonism of CRF-dependent inflammatory priming.

**Fig 4:**
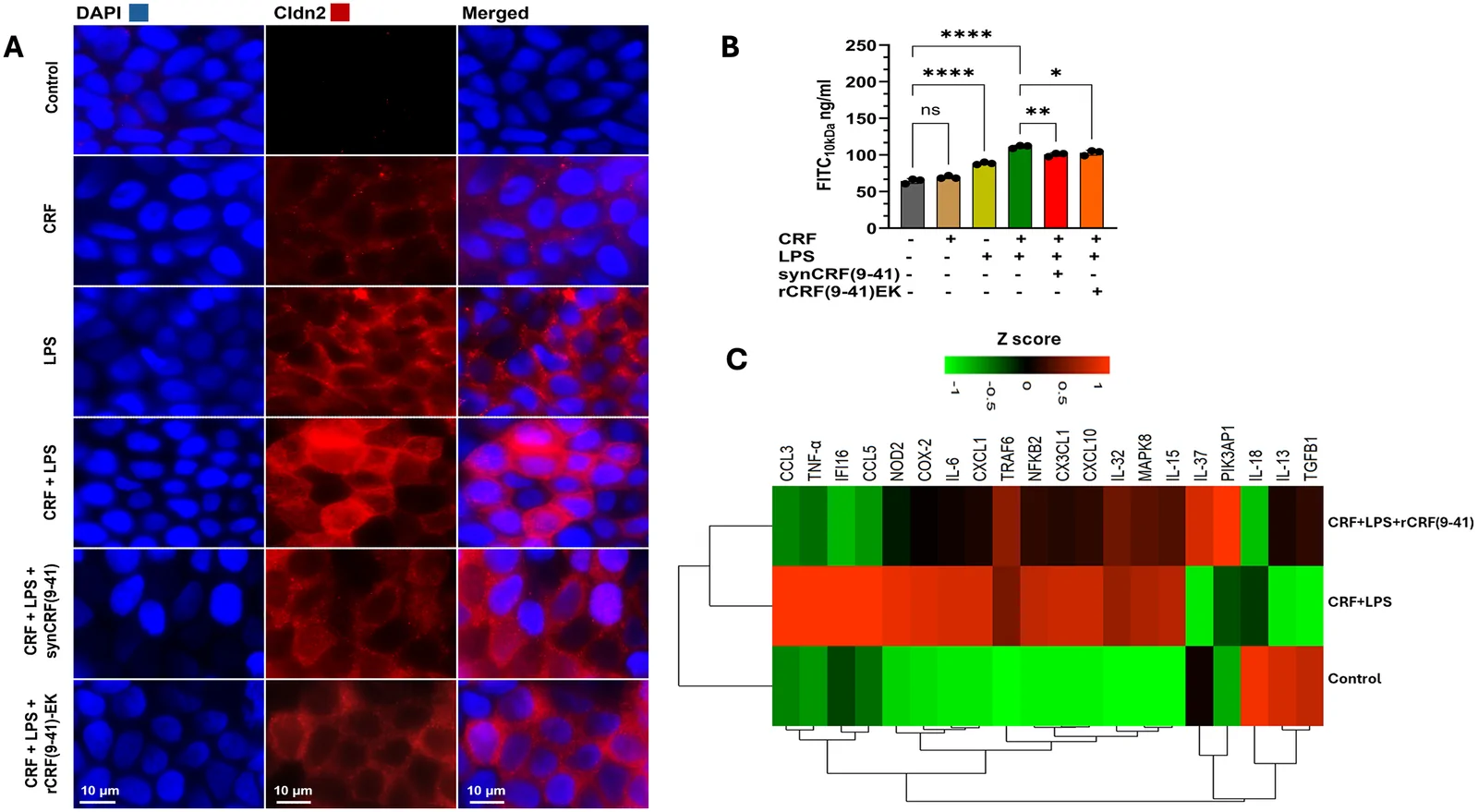
Attenuation of gut epithelial barrier disruption and inflammatory signalling by secreted rCRF(9–41). **(A)** Immunofluorescence analysis of Cldn2 expression in Caco-2 monolayers treated with CRF (100 nM) alone or in combination with LPS (2.5 µg/mL), following pretreatment with either the recombinant [rCRF(9–41)] or synthetic [synCRF(9–41)] antagonist (500 nM). The expression of Cldn2 was detected using a polyclonal primary antibody against Cldn2 and an Alexa Fluor 594 (red)-conjugated secondary antibody. Cell nuclei are counterstained with DAPI (blue) **(B)** FITC-dextran (FD10) concentration in the basal chamber of a transwell permeability assay with Caco2 cell monolayer. The cells were treated with CRF (100 nM) alone or in combination with LPS (2.5 µg/mL), following pretreatment with either rCRF(9-41) or synCRF(9-41) antagonist (500nM). Data are presented as mean ± SEM (n = 3) and One-way ANOVA followed by Tukey’s multiple comparisons test was used for the statistical analysis; *p < 0.05, **p < 0.01, ***p < 0.001, ****p < 0.0001vs. control **(C)** Heatmap of differentially expressed inflammatory genes in Caco-2 cells stimulated with CRF and LPS, in the presence or absence of the recombinant CRF antagonist rCRF(9–41), relative to untreated control groups. Differential gene expression analysis was performed utilizing the DESeq2 package in **R.** Data are visualized as row Z-scores computed from normalized transcript counts to illustrate relative up- or down-regulation across experimental treatments (n = 3). The data underlying this Figure can be found in S1 Data. Raw transcriptomic data can be found under BioProject accession number PRJNA1468903.

To assess effects on epithelial barrier-associated responses, Caco-2 monolayers were treated with CRF, LPS, or CRF + LPS in the presence or absence of CRF antagonists. CRF + LPS co-stimulation markedly increased expression of the tight junction protein claudin-2 (Cldn2), whereas pretreatment with rCRF(9–41) or synthetic antagonist reduced Cldn2 accumulation (Fig 4A). Consistent with these observations, CRF + LPS increased paracellular permeability in FITC–dextran trans well assays, an effect that was attenuated by CRF antagonist pretreatment (Fig 4B).

To further characterize transcriptional changes associated with CRF antagonism, we performed RNA sequencing of Caco-2 cells subjected to CRF + LPS stimulation with or without rCRF(9–41) pretreatment. CRF + LPS exposure induced expression of multiple inflammatory mediators, including IL-6, TNF-α, IL-18, NOD2, and COX-2, while reducing expression of TGFB1, a regulator of epithelial homeostasis (Fig 4C). Pretreatment with rCRF(9–41) attenuated these transcriptional changes, resulting in reduced inflammatory gene expression and partial preservation of TGFB1 levels.

Together, these results demonstrate that rCRF(9–41) secreted by the engineered sensing module retains biological activity in vitro and can modulate CRF-dependent inflammatory and barrier-associated responses under conditions of combined CRF and LPS stimulation.

## Discussion

This study demonstrates that inter-kingdom adrenergic signaling pathways can be rationally repurposed to enable programmable stress hormone sensing in a commensal bacterial chassis by reconstituting the enterohemorrhagic EHEC QseBC two-component system in EcN. We established that host-derived catecholamines can be detected and converted into defined transcriptional and functional outputs. These findings extend previous observations of QseBC-mediated virulence regulation in pathogens [12,24] by showing that adrenergic signaling can be rewired in a non-pathogenic context to yield predictable and tuneable responses.

A central challenge in adapting native bacterial signaling pathways for synthetic purposes is the complexity of their regulatory architecture [22]. Although the *flhDC* operon represents a key QseBC-responsive node in EHEC, its transcriptional output in a heterologous context is constrained by competing global regulators and sigma-factor–dependent control. Our results illustrate that simplification of native promoter architecture through truncation can reduce regulatory interference and unmask signal responsiveness (Fig 2). Further decoupling *flhDC* transcription from σ^28^ availability via σ^70^-dependent promoter replacement yielded a synthetic promoter architecture that produces robust, dose-dependent activation in response to catecholamines. Together, these findings highlight promoter architecture and regulatory insulation as critical determinants of signal fidelity in engineered inter-kingdom sensing systems.

Beyond transcriptional design, our data underscores the importance of expression stoichiometry and translational coupling in two-component system functionality. Disruption of the native operon structure of *q*seBC reduced adrenergic responsiveness (S6A–S6E Fig), consistent with prior reports that coordinated translation is essential for maintaining proper sensor–regulator balance. This observation reinforces a broader principle in bacterial signaling: effective signal transduction depends not only on component identity, but also on their relative expression levels and modes of regulation.

At the molecular level, structure-guided mutagenesis of the QseC periplasmic domain identified residues contributing to catecholamine-responsive signaling (S9A and S9B Fig), providing initial insight into QseC-mediated host signal detection. These findings expand the current understanding of QseC function in EHEC and illustrate how structural modeling can guide the rational engineering of inter-kingdom signaling interfaces. However, future comprehensive structural and direct ligand-binding studies will be required to establish and definitively resolve the molecular basis of QseC-mediated catecholamine sensing and signal transduction.

To demonstrate that adrenergic sensing can be functionally coupled to a biologically relevant output, we linked our optimized module to the regulated secretion of the CRF receptor antagonist peptide rCRF(9–41), utilized here as a model therapeutic payload (Fig 3). In vitro analyses confirmed that the secreted peptide retained robust bioactivity, successfully modulating CRF-dependent inflammatory and barrier-associated responses under concurrent CRF and LPS stimulation (Fig 4). Crucially, these experiments were executed to validate signal transduction and output coupling mechanics rather than to claim clinical efficacy.

A notable limitation of the current in vitro framework is the intrinsic chemical instability and rapid oxidation of catecholamines under co-culture conditions, which constrain the duration and robustness of autonomous sensing-linked output. In addition, secretion levels achieved in the present system remained below those used in purified peptide bioactivity assays; accordingly, conditioned-medium validation of a fully integrated sensing-to-output circuit was not included in this study. Further optimization of circuit sensitivity, basal activity, and secretion efficiency will be required to strengthen end-to-end functional coupling under more demanding assay conditions. Despite these limitations, the present study establishes a modular framework for converting host-associated catecholamine signals into programmable bacterial gene expression and downstream peptide output.

Plasmid-based expression enabled rapid prototyping and stoichiometric optimization of the signaling components used in this study, but long-term maintenance of plasmid-encoded circuits can introduce metabolic burden, evolutionary instability, and unintended basal activity. Future improvements in circuit robustness will likely benefit from chromosomal integration and burden-minimized architectures that better support genetic stability and control of the basal OFF-state. Additional strategies to further tighten the OFF-state may include promoter refinement, incorporation of transcriptional repressors, and circuit-level regulatory mechanisms to reduce background expression and improve signal fidelity. Although further studies will be required to evaluate circuit performance in complex in vivo environments, the framework described here provides a foundation for dissecting inter-kingdom signaling mechanisms and for constructing microbial systems capable of interpreting and responding to diverse host-derived signals.

## Materials and methods

### Construction and expression of the QseBC catecholamine-sensing module

The *qseBC* gene cassette from EHEC was synthesized as a double-stranded DNA fragment (gBlock; Integrated DNA Technologies). The cassette was PCR-amplified using primers listed in S4 Table containing constitutive promoters (J23104, J23106, J23108, J23111, and J23114) from the Anderson promoter library (S3 Table) to enable promoter-strength–dependent tuning of *qseBC* expression. Amplified fragments were cloned into the pBbS8A plasmid backbone using *EcoRI* and *BamHI* restriction sites and transformed into the *E. coli* Top10 (S1 Table). All constructs were sequence verified prior to downstream use (S2 and S5 Tables).

### Transcript profiling of *Escherichia coli* Nissle 1917 expressing EHEC QseBC

To analyze transcriptional changes associated with catecholamine sensing, transcriptomic profiling was performed on EcN expressing the EHEC-derived QseBC system following (−)-norepinephrine (Cat# A7257, Sigma-Aldrich) 100 μM induction. Three independent colonies of wild-type EcN and engineered EcN harboring pBbS8A-J111-*qseBC* (EHEC) were each inoculated into 5 mL of Dulbecco’s modified Eagle medium (11965092, DMEM) supplemented with 25 mM glucose, 0.6% (w/v) casamino acids, and Trace Metal Mix A5 (Cat# 9249, Sigma-Aldrich). Cultures were grown overnight at 37 °C with shaking at 220 rpm, then sub-cultured into freshly supplemented DMEM (Cat# 11965092, DMEM) and grown to mid-log phase (OD₆₀₀ ≈ 0.6). Cells were harvested by centrifugation at 4,000*g*, and total RNA was extracted from cell pellets using the RNeasy Mini Kit (Cat# 74106, Qiagen) according to the manufacturer’s instructions. RNA quality and concentration were assessed prior to sequencing. High-quality RNA samples were submitted to Novogene (NovogeneAIT Genomics) for library preparation and sequencing. Directional mRNA libraries were prepared and sequenced on an Illumina NovaSeq platform using paired-end 150-bp reads (PE150), generating approximately 12 million reads per sample.

Differential gene expression analysis was performed using the DESeq2 package in R. Genes with an adjusted *p* value (padj) < 0.05 and an absolute log_2_fold change ≥ 0.5 were considered significantly differentially expressed.

### Construction and characterization of EHEC flagellar promoter-based catecholamine biosensors

To evaluate the sensitivity of EHEC flagellar promoters to NE/Epi signaling in EcN, selected promoters (*flgBCDEF*, *flgGHIJ*, and *flhDC*) were synthesized as double-stranded gBlocks (Integrated DNA Technologies). Each promoter was cloned upstream of a RFP reporter in the pBbS8A plasmid backbone containing the EHEC-derived *qseBC* regulatory cassette.

To assess the impact of promoter length on transcriptional activity, truncated versions of the *flhDC* promoter (300 and 600 bp upstream of the transcription start site) were generated using primers listed in S4 Table, yielding *flhDC*_EHEC-300_ and *flhDC*_EHEC-600_ variants. These fragments were cloned upstream of the RFP reporter using the same cloning strategy. In addition, a synthetic promoter (SynflhDC) containing both high- and low-affinity QseB binding motifs were designed, synthesized as a gBlock and cloned upstream of the RFP reporter. To further tune promoter responsiveness, RNA polymerase σ^70^/σ^28^ binding motifs were modified using Anderson constitutive promoter variants (J23105, J23111, and J23117), and the corresponding promoter sequences were synthesized as gBlocks incorporating these motifs.

For biosensor characterization, single colonies were grown overnight in LB broth and subsequently sub-cultured into phenol red–free DMEM (Cat# 31053028, DMEM) supplemented with Trace Metal Mix A5 (Cat# 9249, Sigma-Aldrich). Cultures were induced with varying concentrations of norepinephrine (NE), epinephrine (Epi) (25 μM, 50 μM, 100 μM) and AI-3 [(3,5 dimethylpyrazin-2-ol-) 25 μM, 50μM, 100 μM)] and incubated under microaerobic conditions in sealed plate or sealed tube at 37 °C for 16 h. Fluorescence was quantified using a BioTek Synergy H1 plate reader (Agilent) with excitation/emission wavelengths of 535/600 nm. For flow cytometric analysis, cells were washed with 1× phosphate-buffered saline (PBS), resuspended, and analyzed using a BD Accuri C6 flow cytometer (BD Biosciences). All experiments were performed in biological triplicates, and fluorescence values were normalized to optical density at 600 nm (OD₆₀₀).

For decoupling studies, *qseB*_EHEC_ and *qseC*_EHEC_ were individually cloned under the control of the constitutive promoters J23104 and J23111, respectively. Plasmid constructs were introduced into electrocompetent EcN by electroporation. For analysis of QseB and QseC protein expression, EcN cultures were harvested by centrifugation at 4,000*g*, and cell pellets were lysed by heat treatment at 95 °C for 5 min. Crude lysates were resolved by SDS–PAGE and stained overnight with InstantBlue protein stain (Cat# ab119211, Abcam). Gels were washed twice with Milli-Q water and imaged to confirm protein expression. To assess sensing activity, a *flhDC*_EHEC_-_300–RBS–RFP_ reporter cassette was cloned upstream of *qseB*_EHEC_ and *qseC*_EHEC_ in the decoupled constructs. Sensor sensitivity was then evaluated in response to norepinephrine at concentrations of 50 µM and 100 µM, as described above.

### QseC mutant design and functional characterization

To investigate the structural determinants of ligand recognition by the QseC periplasmic sensor domain, a structural model of QseC was generated using AlphaFold2. Molecular docking was then performed to identify potential interactions between sensor domain QseC (34-156) and the small-molecule ligands NE and Epi. Docking simulations were carried out using AutoDock 4.5 software [34]. For each ligand, 100 docking poses were generated using default parameters, and the conformation with the lowest predicted binding energy was selected for further interaction analysis. The docking results identified residues within the QseC that are predicted to contribute to catecholamine binding, including Thr72, Asn74, Asp83, and Asp101. These residues were selected for experimental validation based on their predicted proximity to the ligand and conservation across QseC homologs. Site-directed mutagenesis was carried out to individually substitute the selected residues using the QuikChange Lightning Multi Site-Directed Mutagenesis Kit (Cat# 210513, Agilent), following the manufacturer’s instructions. All mutant constructs were verified prior to functional analysis. The biosensor performance of QseC variants was evaluated using the catecholamine-responsive genetic circuit described above, enabling assessment of the contribution of individual residues to NE and Epi sensing.

### Membrane fractionation and analysis of QseC variants

Membrane fractionation was performed to assess localization of QseC variants and exclude gross protein misfolding or defective membrane association. Engineered *E. coli* Nissle 1917 (EcN) strains expressing wild-type QseBC, point mutants (N74A, D83A, D101A), or an empty-vector control (EcN-EV) were grown to mid-log phase (OD _600_ ~0.6) and harvested (5,000*g*, 10 min, 4 °C). Cell pellets were resuspended in lysis buffer (50 mM Tris-HCl pH 8.0, 150 mM NaCl, 1 mM EDTA, protease inhibitor cocktail) and lysed by probe sonication on ice (40% amplitude; 10 cycles of 10 s ON/30 s OFF). Cell debris was removed and crude membrane fractions were isolated by ultracentrifugation, for 30 min, 4 °C. Membrane pellets were solubilized in lysis buffer containing 1% Triton X-100 for 30 min at room temperature and clarified by ultracentrifugation, for 30 min, 4 °C. Solubilized membrane fractions were analyzed by SDS–PAGE and InstantBlue staining. Comparable abundance of membrane-associated QseC (~50.2 kDa) across wild-type and mutant strains suggested that the reduced signaling phenotypes were unlikely to arise from major defects in protein stability or membrane localization.

### Protein expression, purification and enterokinase digestion

To generate the NE and Epi sensing and response construct, the CRF receptor antagonist peptide rCRF(9–41) encoded gene was synthesized from GenScript and placed under the control of the engineered catecholamine-responsive promoter SynflhDC. To assemble the gene cassette, the rCRF(9–41) peptide was fused to YebF, an *Escherichia coli* K-12 protein known for efficient secretion into the extracellular environment [35]. An enterokinase (EK) recognition site (Asp-Asp-Asp-Asp-Lys) was inserted between YebF and rCRF(9–41) to allow proteolytic cleavage and activation of the peptide. DNA fragments encoding the SynflhDC promoter, the YebF–EK–rCRF(9–41) fusion sequence, and the pBbS8A–J104–qseBC plasmid backbone were assembled using Gibson Assembly with the HiFi DNA Assembly Master Mix (Cat# E2621L, New England Biolabs), following the manufacturer’s instructions. The resulting construct was transformed into EcN to generate a strain capable of sensing NE/Epi and secreting rCRF(9–41). To evaluate secretion of rCRF(9–41), EcN harboring the SynflhDC–YebF–EK–rCRF(9–41) construct was cultured in Dulbecco’s Modified Eagle Medium (Cat# 11965092, DMEM) supplemented with Trace Metal Mix A5. Norepinephrine was added to the culture medium at final concentrations of 25 and 50 μM. Cultures were incubated overnight at 37 °C under microaerophilic conditions in a sealed tube. Cells were subsequently pelleted by centrifugation, and the culture supernatant was concentrated using Vivaspin 500 centrifugal concentrators (Cat# VS15T92, 3 kDa MWCO; Sartorius). Expression of the fusion protein and release of the recombinant CRF antagonist [rCRF(9–41)] were assessed by Western blotting using an anti-His tag antibody. Protein samples were separated by SDS–PAGE and transferred onto a 0.45 μm nitrocellulose membrane (Bio-Rad). Membranes were blocked with 3% bovine serum albumin (BSA) for 1 h at room temperature and incubated overnight at 4 °C with HRP-conjugated anti-His antibodies (MA121315, Invitrogen). After washing with Tris-buffered saline containing 0.1% Tween-20 (TBST), signals were detected using SignalFire Elite ECL reagent (Cat# 12757, Cell Signaling Technology) according to the manufacturer’s instructions. Blots were visualized using the iBright CL1500 Imaging System (Invitrogen).

For bioassay studies, the secreted fusion peptide [YebF–EK–CRF(9–41)] was purified from the culture supernatant using Ni Sepharose Excel resin (Cytiva). The purified protein was buffer-exchanged into 20 mM Tris-HCl and digested with recombinant enterokinase (Merck) at a ratio of 1 U per 50 μg of protein. Digestion was performed either overnight at room temperature or for 1 h at 37 °C.

### Quantification of secreted rCRF(9–41)-HisTag

Single colonies of the NE-responsive SynflhDC-based EcN biosensor strain carrying the rCRF(9–41)-HisTag output cassette were grown overnight in LB broth at 37 °C. Overnight cultures were sub-cultured into phenol red–free DMEM (Cat. No. 31053028, Gibco/Thermo Fisher Scientific) supplemented with Trace Metal Mix A5 (Cat. No. 92949, Sigma-Aldrich). Cultures were induced with 50 μM norepinephrine (NE) and incubated under microaerobic conditions in sealed tubes at 37 °C for 16 hours. Following induction, cultures were centrifuged, and clarified supernatants were collected.

Secreted rCRF(9–41)-HisTag levels were quantified using a His-tag ELISA Kit (Abcam, ab322565) according to the manufacturer’s instructions. Peptide concentrations were calculated from a standard curve and expressed as nanomolar values.

### Cell culture and differentiation THP-1 cell lines

THP-1 (human monocytic) cells were cultured in RPMI-1640 (Cat# SH40007.01, Cytiva) medium supplemented with 10% fetal bovine serum (Cat# S1810, Atlantis Bioscience) and maintained in suspension at 37 °C with 5% CO_2_, with medium changes every 2–3 days. Differentiation into macrophage-like cells was induced by treating THP-1 cells with 100 ng/mL phorbol 12-myristate 13-acetate (P8139, Sigma-Aldrich) for 48–72 hours. To examine the effects of the CRF antagonist, the medium was replaced with fresh RPMI-1640 without FBS prior to stimulation. Differentiated THP-1 macrophages were then stimulated with 100 nM CRF, 1.0 µg/mL lipopolysaccharide (Cat# L4391, LPS-Sigma-Aldrich), or a combination of CRF (100 nM) and LPS (1.0 µg/mL). In parallel experiments, macrophages were pretreated for 1 hr with either the enterokinase digested recombinant enterokinase-digested fusion peptide [YebF-EK-CRF (9−41)-500nM] or the synthetic CRF antagonist [synCRF(9-41)-500 nM] prior to CRF + LPS stimulation. Levels of IL-6 and IL-1β in the culture supernatants were quantified using Human IL-6 and IL-1β ELISA Kit (Cat# ab178013 and ab46052, Abcam) according to the manufacturer’s instruction. Synthetic CRF and synCRF(9–41) peptides used in these experiments were obtained from GenScript.

### Cell culture of Caco-2 and RNA-Seq analysis of treatment groups

Caco-2 cells were cultured in DMEM supplemented with 15% Fetal Bovine Serum (FBS) and 1× Penicillin–Streptomycin (PS) solution. Cells were seeded in 6-well plates and maintained at 37°C in a 5% CO_2_, with regular media changes until confluence is reached. To investigate the effect of the [rCRF(9-41)] antagonist, the culture medium was replaced with fresh FBS-free DMEM. Confluent Caco-2 cells were stimulated with a combination of CRF (100 nM) and LPS (2.5 μg/mL). In parallel, before CRF and LPS stimulation, an additional set of Caco-2 cells was pretreated for 1 hour with either enterokinase-digested fusion peptide [YebF-EK-CRF (9-41)-500nM] or synthetic CRF antagonist [synCRF(9-41)-500nM]. Following antagonist treatment, Caco-2 cells were washed once with 1× PBS and RNA was extracted using TRIzol Reagent (Cat# 15596018, Invitrogen) according to an optimized protocol [36]. Briefly, 200 μl of TRIzol Reagent was added to each well and adherent cells were removed by pipetting. Fifty μl of chloroform was then added, and samples were vigorously vortexed for 30 s and placed at room temperature for 5 min. Following centrifugation at 12,000*g* at 4 °C, the aqueous layer containing RNA was carefully transferred to a clean tube and RNA was precipitated using standard isopropanol methods. RNA pellets were reconstituted in DEPC-treated water and stored at −20 °C. RNA from three independent replicates from each group: (1) Control, (2) CRF+ LPS, and (3) CRF+ LPS+ rCRF(9-41) pretreatment was sent for directional mRNA library preparation (NovogeneAIT Genomics). Poly-A enrichment was performed, and libraries were sequenced on the NovaSeq platform. The sequencing was conducted with paired-end 150 bp (PE150) reads, generating approximately 40 million reads (12 Gb) per sample, providing high-depth transcriptome data for downstream analyses. Data analysis was performed using NovoMagic, a cloud-based bioinformatics platform developed by Novogene. In addition, differential gene expression analysis was conducted using the DESeq2 R package. DESeq2 and Z-score normalization was conducted to normalize between the groups.

### Transwell and permeability assay

To perform the FITC-dextran permeability assay, Caco-2 cells were seeded at a density of 2.5 × 10^4^ cells per well in 24-well trans wells (Cat# PIHP01250, Millipore). Thereafter, cells were subjected to polarization with FBS-free DMEM on the apical compartment and FBS-supplement DMEM on the basal compartment. Media change was conducted every 2 days for 18 days. On the final day, a media change was conducted, and cells were stimulated with 100 nM CRF, 2.5 μg/mL LPS, or a combination of CRF (100 nM) and LPS (2.5 μg/mL) on apical chamber and incubated for 24 hours. In another group, cells were pretreated with recombinant CRF antagonist peptide [rCRF(9-41)] or synthetic CRF antagonist [synCRF (9-41)] for 1 hour before stimulation with the CRF and LPS combination and incubated 24 hours.

On the day of the experiment, the culture medium was removed from the apical side and washed with PBS. Thereafter, 100 μg/mL solution of FITC-dextran 10 kDa (Cat# FD10S-100 MG, Sigma-Aldrich) was prepared in PBS and added to the apical compartment (200 μL), while phenol red-free DMEM was added to the basolateral compartment (600 μL). The transwell plates were incubated at 37° C for 2 hours. After incubation, 100 μL samples were collected from the basolateral compartment and replaced with fresh DMEM media to maintain volume consistency. The fluorescence of the samples was measured using a fluorescence plate reader at an excitation wavelength of 485 nm and emission wavelength of 520 nm. We made a standard curve of FITC-Dextran to calculate the FITC-dextran flux. Permeability was calculated based on the fluorescence intensity of FITC-Dextran in the basolateral compartment.

### qRT-PCR for TLR4 expression in THP-1 and Caco-2

Total RNA was extracted from THP-1 and Caco-2 cells using the TRIzol method, as described previously [37,38]. Complementary DNA (cDNA) was synthesized using the HiScript III cDNA Synthesis Kit (Cat# R312-02, Vazyme) according to the manufacturer’s instructions. Briefly, 1 μg of total RNA was treated to remove genomic DNA prior to reverse transcription using the HiScript III enzyme mix at 37 °C. The resulting cDNA was diluted, and quantitative PCR was performed using Luna Universal qPCR Master Mix (Cat# M3003, New England Biolabs) on a CFX Connect Real-Time PCR Detection System (Bio-Rad Laboratories). Relative RNA expression levels were calculated using the 2^−ΔΔCt^ method, with β-actin used as the internal housekeeping gene for normalization [39].

### Immunofluorescence staining

Caco-2 cells were seeded onto sterile glass coverslips placed in 6-well culture plates and grown to the desired confluence. For immunofluorescence staining, culture medium was removed and cells were washed three times with 1× PBS to remove residual medium. Cells were then fixed with 4% paraformaldehyde (Cat# 16005, Sigma-Aldrich) for 10 min at room temperature or overnight at 4 °C. Following fixation, cells were washed three times with PBS and blocked with 1% BSA in PBS for 1 hour at room temperature to reduce nonspecific antibody binding. Cells were subsequently incubated overnight at 4 °C with gentle agitation with a rabbit anti–claudin-2 polyclonal primary antibody (Cat# 51-6100, Thermo Fisher Scientific). After primary antibody incubation, cells were washed three times with PBS and incubated with an Alexa Fluor 594–conjugated anti-rabbit IgG secondary antibody (Cat# 8889, Cell Signaling Technology) for 1 hour at room temperature in the dark. Nuclei were counterstained with 4′,6-diamidino-2-phenylindole (DAPI; NucBlue, Cat# R37605, Invitrogen). Coverslips were mounted onto glass slides using a mounting medium and sealed. Fluorescent images were acquired using a Leica DM4000 B fluorescence microscope under identical exposure settings for all experimental conditions.

### Statistical analysis

All statistical analyses were performed using GraphPad Prism software (GraphPad Software, San Diego, CA). Data are presented as mean ± standard error of the mean (SEM), unless otherwise indicated. Statistical significance was assessed using one-way analysis of variance (ANOVA) followed by Tukey’s multiple-comparison test to compare differences among experimental groups. A *p* value of < 0.05 was considered statistically significant, with significance thresholds defined as *p < 0.05, **p < 0.01, ***p < 0.001, ****p < 0.0001.

## Supporting information

S1 Fig Multiple sequence alignment of QseB homologs from different bacterial species. *Escherichia coli* O157:H7 str. Sakai (Accession Number - BA000007.3, QseB protein_ID - BAB37330.1), *Escherichia coli* Nissle 1917 (Accession Number - CP022686.1, QseB protein_ID - AXY46083.1), *Shigella dysenteriae* strain SC595 (Accession Number - CP118616.1, QseB protein_ID WNT49955.1), *Salmonella enterica* subsp. enterica serovar Typhimurium strain ATCC 14028 (Accession Number - CP117244.1, QseB protein_ID WCT37774.1), *Acinetobacter baumannii* IOMUT 433 (Accession Number - AP014649.1, QseB protein_ID BAP66005.1), *Clostridioides difficile* strain SC084-01-01 (Accession Number - CP132146.1, QseB protein_ID WPV61431.1), Haemophilus influenzae 86-028NP (Accession Number - CP000057.2, QseB protein_ID AAX88763.1), *Klebsiella pneumoniae* str. Kp52.145 (Accession Number - FO834906.1, QseB protein_ID CDO12429.1), *Pseudomonas aeruginosa* IOMTU 133 (Accession Number - AP017302.1, QseB protein_ID BAT65495.1), *Vibrio cholerae* str. BC1071 (Accession Number - LT897798.1, QseB protein_ID SNC57488.1). The colors correspond to similar amino acid across the sequences, and the bar graph indicates the degree of conservation between sequences.
(TIF)

S2 Fig Multiple sequence alignment of QseC homologs from different bacterial species. *Escherichia coli* O157:H7 str. Sakai (Accession Number - BA000007.3, protein_ID (BAB37331.2), *Escherichia coli* Nissle 1917 (Accession Number - CP022686.1, QseC protein_ID AXY46084.1), *Shigella dysenteriae* strain SC595 (Accession Number - CP118616.1, QseC protein_ID WNT49956.1), *Salmonella enterica* subsp. enterica serovar Typhimurium strain ATCC 14028 (Accession Number - CP117244.1, QseC protein_ID WCT37773.1), *Acinetobacter baumannii* IOMUT 433 (Accession Number - AP014649.1, QseC protein_ID BAP66006.1), *Haemophilus influenzae* 86-028NP (Accession Number - CP000057.2, QseC protein_ID AAX88762.1), *Klebsiella pneumoniae* str. Kp52.145 (Accession Number - FO834906.1, QseC protein_ID CDO12428.1), *Pseudomonas aeruginosa* IOMTU 133 (Accession Number - AP017302.1, QseC protein_ID BAT68051.1), *Vibrio cholerae* str. BC1071 (Accession Number - LT897798.1, QseC protein_ID SNC58272.1). The colors correspond to amino acid identity, and the bar graph indicates the degree of conservation between sequences.
(TIF)

S3 Fig Transcriptomic and regulatory network analysis of flagellar gene expression in response to NE. **(A)** Volcano plot showing the differentially expressed genes (DEGs) between the different groups (EcNWT-NE vs. EcNWT, EcNqseBC-EHEC-NE vs. EcNWT and EcNqseBC/EHEC-NE vs. EcNWT-NE), with up-regulated genes highlighted in red and down-regulated genes in green. Differential expression analysis was performed in R using the DESeq2 package with DEGs selected based on padj < 0.05 and |log2(FoldChange)| > 0. **(B)** Expression profile of flagellar genes clusters in EcNqseBC/EHEC-NE versus EcNWT–NE, demonstrating the impact of heterologous qseBCEHEC expression on NE-responsive flagellar biosynthesis genes. **(C)** Transcriptional regulatory network illustrating flagellar biosynthesis, highlighting the hierarchical control of flagellar operons by key transcriptional regulators and signaling pathways. The network depicts interactions between key transcription factors and their downstream target operons, illustrating how flagellar gene expression is dynamically modulated in response to external stimuli. **(D)** Multiple sequence alignment of the flhDC operon promoters from *E. coli* O157:H7 (EHEC) and *E. coli* Nissle 1917 (EcN). (E-F) Evaluation of the sensing activity of the flagellar promoters flgBCDEFEHEC and flgGHIJEHEC in the presence of 100 µM NE. The data underlying this Figure can be found in S2 Data. Raw transcriptomic data for S3A Fig can be found under BioProject accession number PRJNA1467173. Created in BioRender. Aggarwal, N. (2026) https://BioRender.com/z371w74.
(TIF)

S4 Fig NE-dependent qseBC activation remodels the EcN-qseBC-EHEC transcriptome. **(A)** Volcano plot of differentially expressed genes in NE-induced versus uninduced EcN-qseBC-EHEC. Using padj ≤ 0.05, 950 genes were upregulated and 897 downregulated following NE induction. Dashed lines indicate fold-change thresholds. **(B)** Heatmap illustrating the expression profiles of selected genes involved in flagellar assembly, motility, chemotaxis, and quorum sensing, revealing coordinated transcriptional regulation following NE-mediated QseBC activation. Expression values are shown as row Z-scores (red, increased; blue, decreased). Raw transcriptomic data can be found under BioProject accession number PRJNA1467173.
(TIFF)

S5 Fig Functional impact of decoupling translational coupling within the *qseBC* operon. **(A)** Genetic architecture depicting native translational coupling of qseB and qseC within the qseBC operon **(B)** Decoupling of qseB and qseC through independent expression driven by the synthetic promoters J23104 (qseB) and J23111 (qseC) promoter. **(C)** Expression levels of QseB and QseC were confirmed by SDS-PAGE, which displayed distinct bands corresponding to their respective molecular weights of 24.67 kDa for QseB and 50.28 kDa for QseC. **(D, E)** Genetic construct with the flhDCEHEC-300 promoter fused to a reporter under decoupled qseB/qseC expression and NE induction (50μM and 100μM) demonstrates loss of signal transmission and NE-sensing activity. Data are presented as mean ± SEM (*n* = 3). Statistical significance was determined using one-way ANOVA followed by Tukey’s multiple comparisons test. ns, not significant compared with the uninduced control (0 µM NE). The data underlying this Figure can be found in S2 Data. Created in BioRender. Aggarwal, N. (2026) https://BioRender.com/z371w74.
(TIFF)

S6 Fig Characterization of the Anderson synthetic promoter library in EcN and the laboratory strain *E. coli* MG1655. Each promoter was transcriptionally fused to a RFP reporter with the promoter activity quantitatively assessed by measuring RFP fluorescence in cells grown in M9 minimal medium. Fluorescence values were normalized to optical density at 600 nm (OD₆₀₀) to account for differences in cell density, enabling direct comparison of promoter activity between strains. The data underlying this Figure can be found in S2 Data and S1 Raw Images. Created in BioRender. Aggarwal, N. (2026) https://BioRender.com/z371w74.
(TIFF)

S7 Fig Transcriptional evaluation of low-affinity QseB-binding motifs in synthetic promoter constructs. **(A)** Schematic representation of the low-affinity-only promoter construct, generated by retaining the low-affinity QseB-binding motif upstream of the flhDC synthetic promoter (SynflhDC) architecture. **(B, C)** Promoter activity was quantified using an RFP reporter (RFP/OD600) following stimulation with increasing concentrations of norepinephrine (NE) and epinephrine (Epi). The low-affinity-only construct exhibited limited activation across the tested concentration range, supporting the contribution of combined high- and low-affinity QseB-binding motifs to enhanced catecholamine-responsive transcriptional activation. Data represent mean ± SEM (*n* = 3). The data underlying this Figure can be found in S2 Data. Created in BioRender. Aggarwal, N. (2026) https://BioRender.com/z371w74.
(TIFF)

S8 Fig Growth characterization, dose-response kinetics, and peptide secretion efficiency of engineered EcN. **(A)** Growth curves of wild-type *E. coli* Nissle (EcN-WT) and engineered EcN expressing J23104-qseBC under standard culture conditions, showing comparable growth profiles. **(B, C)** Dose–response analysis of norepinephrine (NE) and epinephrine (Epi) demonstrating concentration-dependent activation of the SynflhDC reporter, with measurable responses observed from approximately 25 μM. **(D)** Fold induction of SynflhDC promoter activity following exposure to norepinephrine (NE) and AI-3. Promoter activity was quantified as RFP/OD600, normalized to the corresponding untreated control (0 μM) for each analyte, and expressed as fold induction. **(E)** Secreted rCRF(9–41)-HisTag levels were quantified from culture supernatants using a HisTag ELISA assay, demonstrating secretion levels of approximately 65–84 nM following NE induction under the tested culture conditions in DMEM minimal medium after 14 h incubation. Data are presented as mean ± SEM (*n* = 3). Statistical significance was determined using one-way ANOVA followed by Tukey’s multiple-comparison test (*P < 0.05, **P < 0.01, ***P < 0.001, ****P < 0.0001 versus uninduced control (0 μM NE). The data underlying this Figure can be found in S2 Data.
(TIFF)

S9 Fig Structural modelling and site-directed mutagenesis of key binding residues in the QseC sensor domain. **(A, B)** AlphaFold-based structural modeling of QseC highlighting the predicted periplasmic sensor domain (residues 34–156). Docking of epinephrine (Epi) and norepinephrine (NE) to the QseC periplasmic domain identified shared candidate interaction residues, including Thr72, Asn74, Asp83, and Asp101. **(C–G)** Site-directed mutagenesis and reporter analysis showed that the QseC sensor-domain mutant N74A markedly reduced catecholamine-responsive signaling, whereas D83A and D101A produced partial effects and T72A showed no detectable effect. AI-3-responsive signaling was further assessed in wild-type QseC and the N74A mutant following stimulation with 50 μM and 100 μM AI-3. The QseC (N74A) variant showed reduced reporter activation compared with wild-type QseC (WT), particularly following stimulation with 100 μM AI-3. UI indicates uninduced. Data are presented as mean ± SEM (*n* = 3). One-way ANOVA followed by Tukey’s multiple comparisons test was used for the statistical analysis; *p < 0.05, **p < 0.01, ***p < 0.001, ****p < 0.0001 vs. uninduced (0µM NE). The data underlying this Figure can be found in S2 Data.
(TIFF)

S10 Fig Membrane localization analysis of QseC variants. SDS–PAGE characterization of 1% Triton X-100–solubilized membrane fractions isolated from EcN strains expressing wild-type QseC (EcN-QseBC) or the indicated point-mutant variants (N74A, D101A, and D83A). An empty-vector strain (EcN-EV) served as a negative control. Comparable abundance of QseC (arrowhead, ~50.2 kDa) across wild-type and mutant samples suggests that the reduced signaling phenotypes are unlikely to arise from gross protein misfolding or defective membrane localization. The data underlying this Figure can be found in S1 Raw Images.
(TIFF)

S11 Fig EcN-secreted and purified rCRF(9–41) modulates CRF-induced TLR4 expression in THP-1 macrophages and Caco-2 cells. **(A, B)** qRT–PCR analysis of TLR4 expression in THP-1-derived macrophages and Caco-2 cells stimulated with CRF (100 nM) following pretreatment (500 nM) with recombinant rCRF(9–41), a synthetic antagonist control [synCRF(9–41)], or a matched purification-process control generated from YebF lacking the rCRF cargo and processed under identical secretion, purification, and enterokinase-treatment conditions. Data represent mean ± SEM (*n* = 3) and were analyzed using one-way ANOVA with Tukey’s multiple-comparison test (*P < 0.05, **P < 0.01, ***P < 0.001, ****P < 0.0001). The data underlying this Figure can be found in S2 Data.
(TIFF)

S12 Fig Schematic illustration of the TLR4 signaling cascade, highlighting both the MyD88-dependent and MyD88-independent (TRIF-dependent) pathways. Activation of these pathways leads to downstream signaling events that culminate in the induction of pro-inflammatory gene expression and inflammatory mediator production. Created in BioRender. Aggarwal, N. (2026) https://BioRender.com/z371w74.
(TIFF)

S1 Table Bacterial strains used in the study. (XLSX)

S2 Table Plasmids and genetic constructs used in the study. (XLSX)

S3 Table Anderson constitutive promoter library used in the study. (XLSX)

S4 Table Primers used in the study. (XLSX)

S5 Table DNA sequences of constructs used in this study. (XLSX)

S1 Data Numerical data for main figures in this study. (XLSX)

S2 Data Numerical data for supplementary figures in this study. (XLSX)

S1 Raw Images Original uncropped gel images and blots. (PDF)

## Abbreviations

ANOVA: analysis of variance
BSA: bovine serum albumin
cDNA: Complementary DNA
CRF: corticotropin-releasing factor
Epi: epinephrine
FBS: Fetal Bovine Serum
HPA: hypothalamic–pituitary–adrenal
NE: norepinephrine
PBS: phosphate-buffered saline
PS: Penicillin–Streptomycin
RFP: red fluorescent protein
SEM: standard error of the mean
TLR4: Toll-like receptor 4
