# Supplementary material for: Engineering inter-kingdom adrenergic signaling in commensals couples host stress hormone sensing to programmable biological outputs
Source: PLoS Biol. 2026 Aug 25;24(8):e3003926. doi: 10.1371/journal.pbio.3003926 (PMC13505935; doi:10.1371/journal.pbio.3003926)

Fig 3G

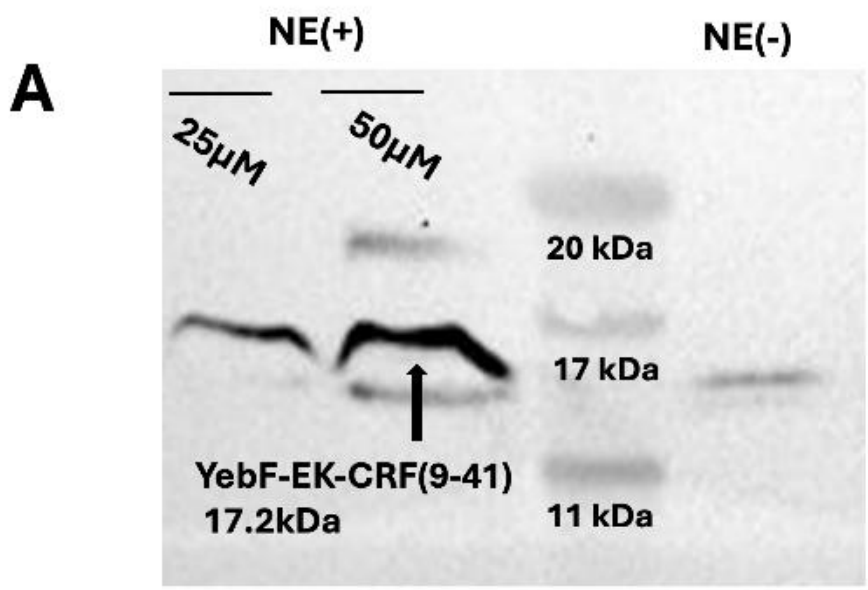

Uncropped Gel image

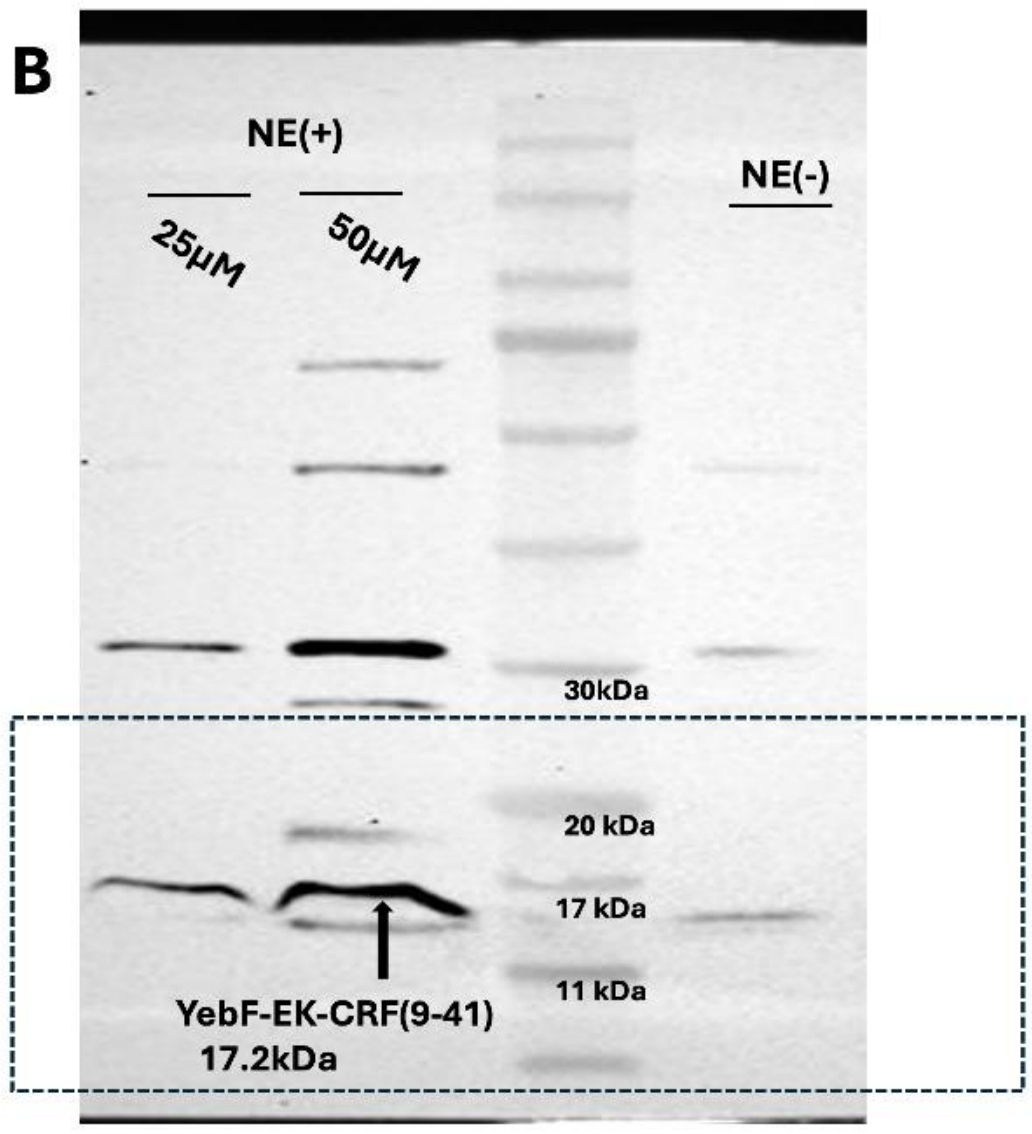

Fig 3H

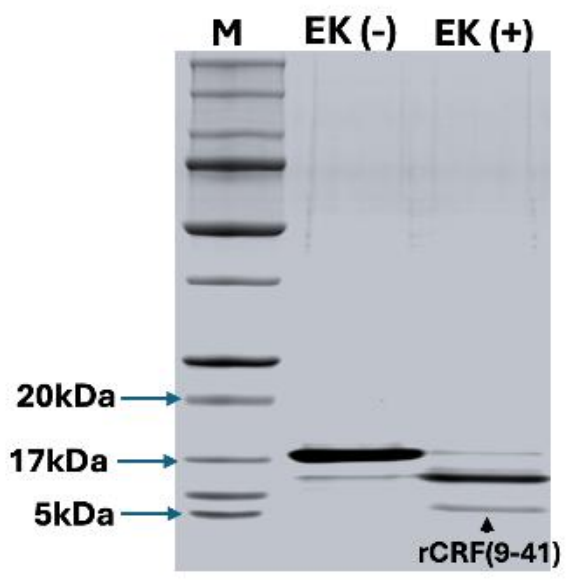

Uncropped Gel image

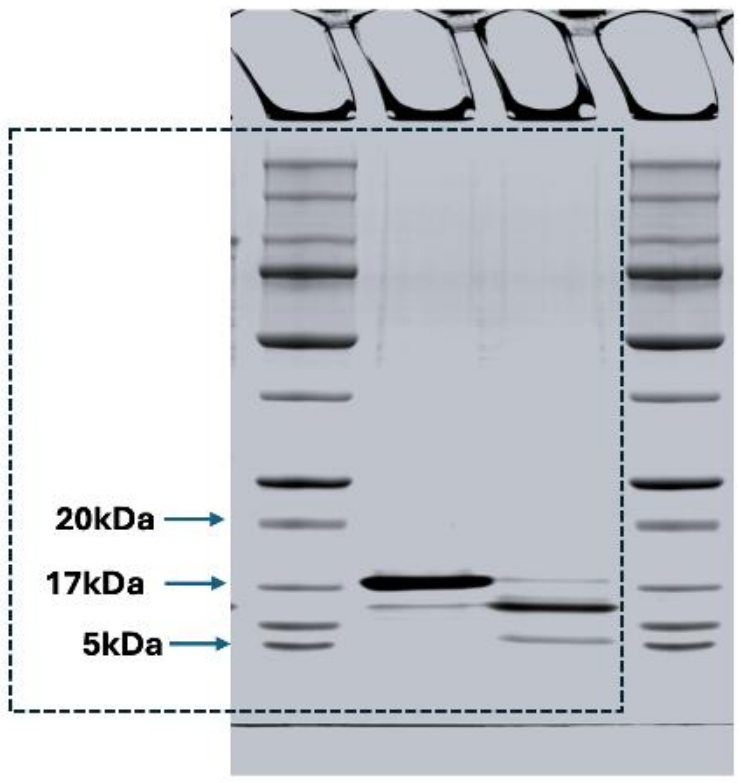

Supplementary Figure 10

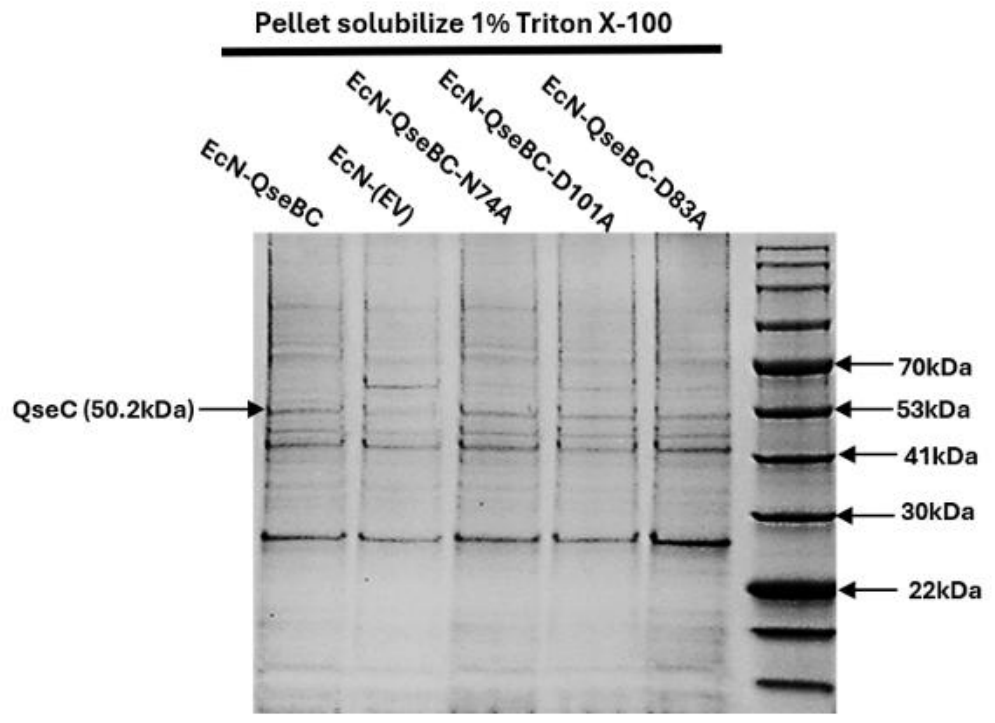

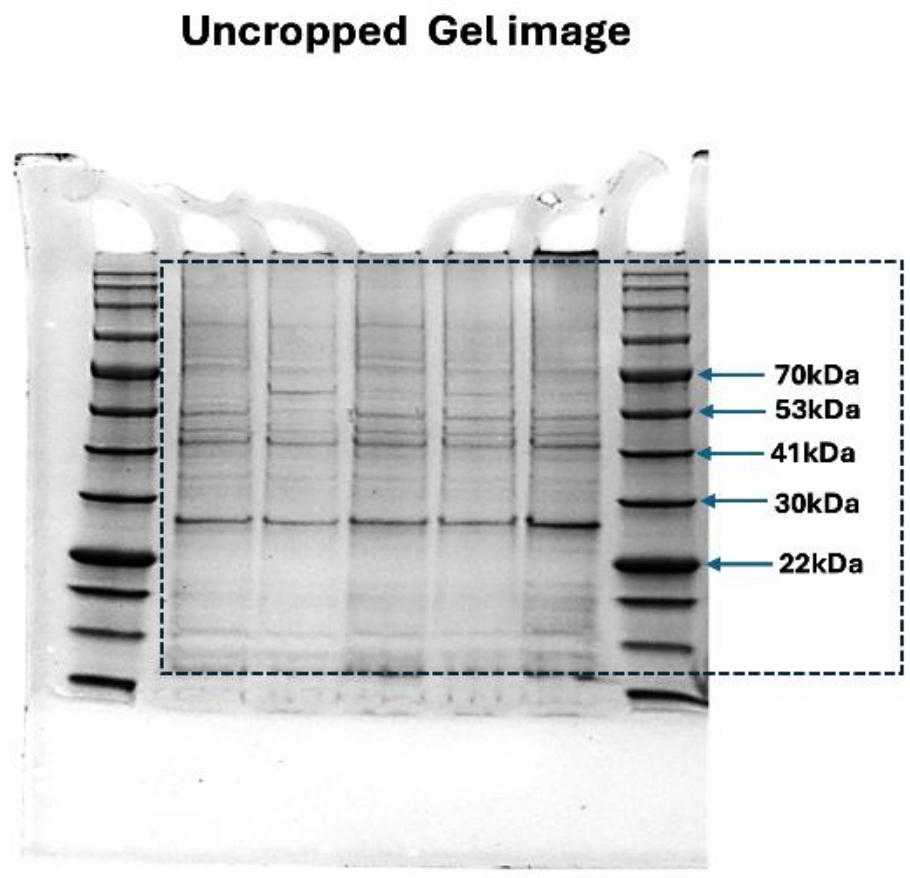

Supplementary Figure 6C

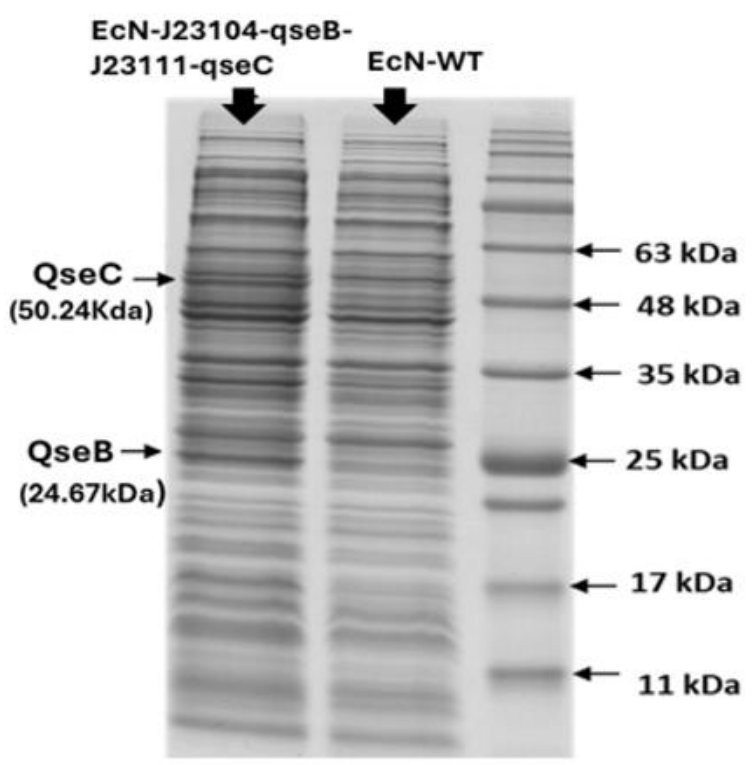

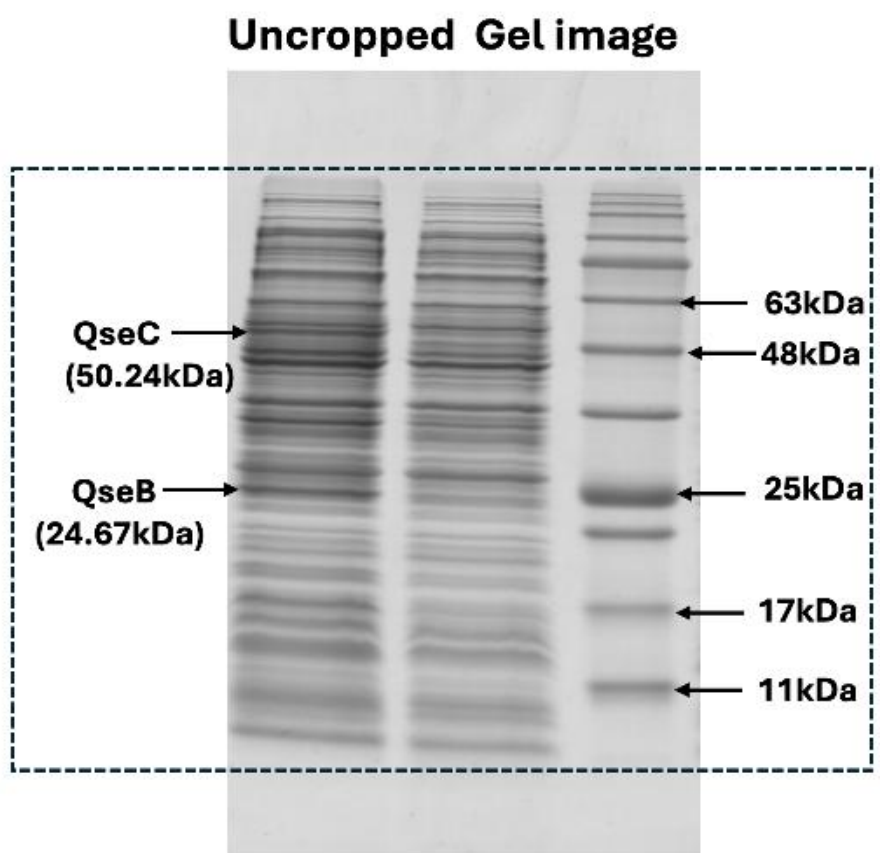

Supplement: Supplementary file 1 [file pbio.3003926.s020.pdf]
